# Genome-Wide Identification and Expression Profiling of the *WOX* Gene Family in *Citrus sinensis* and Functional Analysis of a *CsWUS* Member

**DOI:** 10.3390/ijms22094919

**Published:** 2021-05-06

**Authors:** Faiza Shafique Khan, Ren-Fang Zeng, Zhi-Meng Gan, Jin-Zhi Zhang, Chun-Gen Hu

**Affiliations:** Key Laboratory of Horticultural Plant Biology (Ministry of Education), College of Horticulture and Forestry Science, Huazhong Agricultural University, Wuhan 430070, China; faizakhan@webmail.hzau.edu.cn (F.S.K.); renfzeng@mail.hzau.edu.cn (R.-F.Z.); zhimenggan@webmail.hzau.edu.cn (Z.-M.G.)

**Keywords:** *Citrus sinensis*, *CsWOXs*, transcription factor, *CsWUS*

## Abstract

*WUSCHEL*-related homeobox (*WOX*) transcription factors (TFs) are well known for their role in plant development but are rarely studied in citrus. In this study, we identified 11 putative genes from the sweet orange genome and divided the citrus *WOX* genes into three clades (modern/WUSCHEL(WUS), intermediate, and ancient). Subsequently, we performed syntenic relationship, intron-exon organization, motif composition, and *cis*-element analysis. Co-expression analysis based on RNA-seq and tissue-specific expression patterns revealed that *CsWOX* gene expression has multiple intrinsic functions. *CsWUS* homolog of *AtWUS* functions as a transcriptional activator and binds to specific DNA. Overexpression of *CsWUS* in tobacco revealed dramatic phenotypic changes, including malformed leaves and reduced gynoecia with no seed development. Silencing of *CsWUS* in lemon using the virus-induced gene silencing (VIGS) system implied the involvement of *CsWUS* in cells of the plant stem. In addition, CsWUS was found to interact with CsCYCD3, an ortholog in *Arabidopsis* (AtCYCD3,1). Yeast one-hybrid screening and dual luciferase activity revealed that two TFs (*CsRAP2.12* and *CsHB22*) bind to the promoter of *CsWUS* and regulate its expression. Altogether, these results extend our knowledge of the *WOX* gene family along with *CsWUS* function and provide valuable findings for future study on development regulation and comprehensive data of *WOX* members in citrus.

## 1. Introduction

Homeobox transcription factors (TFs) containing homeodomain proteins are separated into 14 families, including the *WUSCHEL* -related homeobox (*WOX*) TF family [1,2,3]. The domain features a helix–loop–helix–turn–helix (HTH) structure that contains 60–66 amino acids crucial for specific functions in plants [4,5,6]. Two alpha-helices are intricately associated with DNA by a short turn, defined as the HTH motif [7]. WOX proteins usually contain a strongly conserved homeodomain that is highly specific to DNA binding proteins and may act as repressors or activators [3,8]. Homeodomain proteins are widely identified in monocot and dicot plants [9]. The model plant *Arabidopsis* contains 15 *WOX* members, which are divided into three clades based on their evolutionary relationship: modern/WUS, intermediate, and ancient clades [9]. *Arabidopsis WOX* members have been extensively studied, and their orthologs have exhibited diverse functions in numerous development processes [10,11,12,13], along with rice and maize [8,12]. Besides *Arabidopsis*, genome-wide studies have also been conducted on the *WOX* gene families of woody plants such as walnut [10], physic nut [14], grapes [15,16], peach, pear, apricot [17], coffee [18], and poplar [19]. In these plants, *WOX* genes are involved in vital regulatory networks that link the developmental mechanisms in plants, including shoot apical meristem, lateral organ development, plant stem cell maintenance, and floral determinacy [5,20,21,22].

In *Arabidopsis*, *AtWUS* and *AtWOX1* determine floral meristem identity and maintenance [23,24,25]. *AtWOX2*, *AtWOX6*, *AtWOX8*, and *AtWOX9* regulate ovule development and enable the development of cotyledon boundaries, eggs, and zygotes [26]. *AtWOX3* targets pathways that promote flower organ primordia and leaf margin development [27]. *AtWOX7* plays an important role in lateral root development and sugar (sucrose and glucose) status in *Arabidopsis* [28]. *AtWOX11/AtWOX12* is involved in cell fate transition and root organogenesis [29]. Some *WOX* genes in *Arabidopsis* are involved in hormone signaling transduction pathways. For example, *AtWOX4*, *AtWOX5*, and *AtWOX11* regulate auxin signaling that determines lateral organ and apical root growth [19,20,30]. *WOX1* homologs have been demonstrated to control leaf blade outgrowth in *Zea mays*, *Petunia hybrida*, *Medicago truncatula*, and *Nicotiana tabacum* [5,31,32]. Leaf blade outgrowth is controlled by the WOX domain [33]. Recently, 12 WOX proteins were identified in walnut; *JrWOX3a* and *JrWOX3b* enable leaf development. *PpWUS* and *PpWOX5* regulate embryo development in *Pinus pinaster* [3,10]. In Norway spruce, *PaWOX3* promotes lateral organ outgrowth in conifers [34]. In recent years, several previous studies reported that *WOX* genes respond to abiotic stresses and hormone treatment, including *Oraza sativa*, *Gossypium*, and *Brassica napus* [8,23,35,36,37]. However, the response of *WOX* genes during abiotic stress has not been studied adequately in citrus.

Several previous studies indicated that the *WUS* gene is one of the most important genes in the *WOX* gene family and involved in numerous important developmental processes including size of shoot meristem, somatic embryo, as well as adventitious shoot and lateral leaf formation [5,38,39]. For example, *AtWUS* is crucial for shoot apical meristem maintenance to replace the function of *WOX1* and *PRESSED FLOWER* (*PRS)* [5]. The ectopic overexpression of *AtWUS* in tobacco is involved in stem cell fate and lateral leaf formation [40]. In *Medicago truncatula*, the *AtWUS* homolog *HEADLESS* (*HDL*) is also involved in leaf development [31]. *WUS* gene functions downstream of the *CLAVATA3* (*CLV3*) signaling pathway [41]. The *WUS* gene is expressed in the organizing center and enhances *CLV3* expression in stem cells. Likewise, *CLV3* negatively regulates meristem size by suppressing *WUS* expression [42,43]. *WUS* interacts with *CYCLOIDEA 2 (CYC2)* and regulates reproductive organ development (ovary, stigma, and style) in *Chrysanthemum morifolium* [44]. In the embryonic columella, *WOX5* and *CYCD3;3/CYCD1;1* facilitate cell proliferation, and *CYCD3* plays a major role in normal cell division [45]. The *WUS* orthologs *STERILE AND REDUCE TILLERING* (*MOC3/SRT1*) and *TILLERS ABSENT1/MONOCULM 3* are involved in bud formation and female fertility of rice [46]. The *WUS* gene regulates histone acetylation and interferes with HISTONE DEACETYLASE (HDAC) activity, which stimulates the auxin signaling pathway in stem cells [38]. In addition, *WUS* expression is indirectly repressed by *AGMOUS* (*AG*) and stimulates expression of the zinc finger TF C2H2 type (*KNUCKLES*), which in turn suppresses *WUS* expression directly or indirectly involved in the maintenance of floral meristem cells [5]. Thus far, functional characterization of *WUS* genes has been studied in other plants but is rarely studied in citrus.

The above-mentioned studies report that *WOX* TFs primarily affect plant development by regulating the expression of downstream genes. Notably, WOX protein cloning and functional analyses were predominantly focused on model plants such as *Arabidopsis.* However, there have been relatively few studies on the regulatory and genetic development role of the *WOX* family in citrus. The availability of the citrus genome database gives us a valuable genetic resource to study specific sweet orange genes [47,48]. A thorough screening of the citrus database allowed us to find the evolutionary, regulatory, and developmental role of *WOX* orthologs in sweet orange. In the current study, we identified 11 putative *WOX* members in *Citrus sinensis*. Tissue-specific expression patterns and co-expression profiles of *CsWOXs* under water deficit and floral inductive conditions were comprehensively studied. The subcellular localization, transactivation activity, and DNA-binding ability confirmed that *CsWUS* may be a TF. Moreover, *CsWUS* overexpression and the virus-induced gene silencing (VIGS) assay revealed new insights into floral organ development, stem cell activity, and leaf development in citrus. In addition, yeast two-hybrid assays and DNA-protein interactions confirmed the complex involvement of CsWUS in developmental regulatory networks. Our data provide an evolutionary, co-expression, and spatial expression analysis of the *WOX* gene family and new perspectives that contribute to the function of the *WOX* family in citrus.

## 2. Results

### 2.1. Genome-Wide Identification and In Silico Subcellular Localization Prediction of CsWOX Gene Family

To identify citrus *WOX* genes, *Arabidopsis* and rice WOX proteins were used as queries and all resulting sequences were retrieved from the sweet orange database (http://citrus.hzau.edu.cn/cgi-bin/orange/blast) using BLASTP. After removing sequence redundancies of the same protein, a total of 11 potential WOX proteins were identified as being allied with *CsWOX* proteins (Table 1). We further confirmed that all of these CsWOX proteins contained the homeodomain (PF00046 and SM00389). The 11 putative WOX proteins corresponding to the gene were named according to their physical location (from top to bottom) on chromosomes 1–8 (Table 1). Notably, one gene (*CsWOX10*) is located on an unknown chromosome. The coding sequence (CDS) length of *CsWOX* genes varied from 582 bp (*CsWOX7*) to 1104 bp (*CsWOX2*), encoding polypeptides of 193–367 amino acids in length, with a predicted molecular weight range of 15,437.5–40,473.1 Da and a theoretical isoelectric point (pI) ranging from 5.4 to 11.5 (Table 2). In addition, the subcellular localization of *CsWOX* proteins was predicted (Table 2). The 3D structure of proteins was also predicted (Supplementary Figure S1). The predicted locations of three CsWOX proteins (CsWUS, CsWOX3, and CsWOX5) were found to be nuclear localized. The remaining members of CsWOX were projected to be localized in chloroplast, mitochondria, cytoplasm, or plastid.

### 2.2. Phylogenetic Analysis and Gene Structure of CsWOX Genes

#### 2.2.1. Phylogenetic Analysis of CsWOX Genes

**Figure 1:**
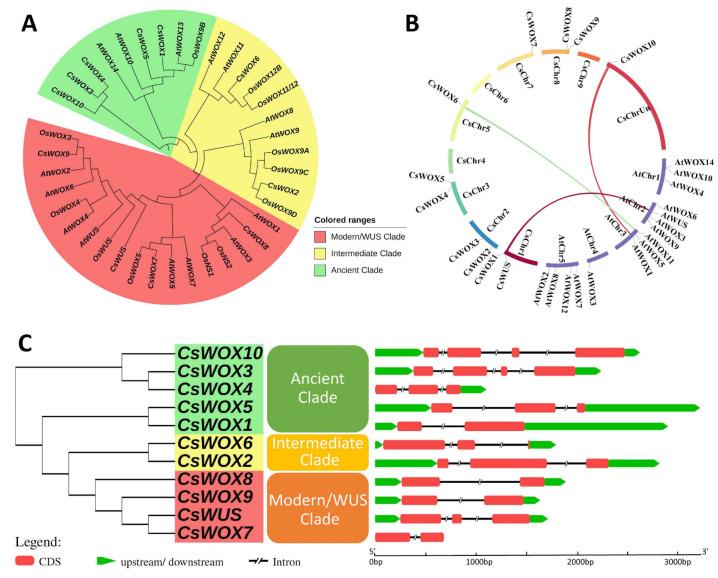
Phylogenetic tree, synteny analysis, exon, and intron distribution of *WOX* genes of citrus. (**A**) Phylogenetic tree of citrus CsWOX; rice (*OsWUS*: AM234746; *OsWOX3*: AM234749; *OsWOX4*: AM234750; *OsWOX5*: AM234751: *OsWOX9A*: Q0JKK6; *OsWOX9B*: AM234755; *OsWOX9C*: AM234752; *OsWOX9D*: AM234753; *OsWOX11/12*: AM234754*; OsWOX12B*: ABF95709; *OsNS2*: AM234748; *OsNS1*: AB218893); and *Arabidopsis* (*AtWUS*: AT2G17950; *AtWOX1*: AT3G18010; *AtWOX2*: AT5G59340; *AtWOX3*: AT2G28610; *AtWOX4* AT1G46480; *AtWOX5*: AT3G11260; AtWOX6: AT2G01500; AtWOX7: AT5G05770; AtWOX8: AT5G45980; AtWOX9: AT2G33880; AtWOX10: AT1G20710; *AtWOX11*: AT3G03660; *AtWOX12*: AT5G17810; *AtWOX13*: AT4G35550; *AtWOX14*: AT1G20700). (**B**) Synteny analysis and chromosomal distribution of *CsWOX* genes; colored bars joining two chromosomal regions represents syntenic regions. Chr, Chromosome. (**C**) Exon-intron distribution. CDS exon indicated by red boxes, upstream and downstream region indicated by green boxes, intron indicated by black line.

To explore the phylogenetic relationship of WOXs between citrus and other model plants, a phylogenetic tree was resolved using 39 WOX family members from *Arabidopsis* (15 genes), rice (13 genes), and sweet orange (11 genes). The phylogenetic distribution showed that all *CsWOX* members were grouped into three clades, modern/WUS, intermediate, and ancient, consistent with previous *WOX* family distribution schemes [2]. The modern/WUS clade was the largest in this phylogenetic tree, containing 18 members: four members from sweet orange, six from rice, and eight from *Arabidopsis*. The intermediate clade was the second largest and included two from sweet orange, six from rice, and four from *Arabidopsis* members. The ancient clade had five members from sweet orange, one from rice, and three from *Arabidopsis*. Additionally, we also explored the orthologous relationships among sweet orange, rice, and *Arabidopsis* WOX families. These included 13 orthologous genes, putative orthologs with sweet orange proposed based on the phylogenetic tree were as follows: *CsWOX5/AtWOX10*, *CsWOX1/AtWOX13*, *CsWOX6/AtWOX11*, *CsWOX2/AtWOX9*, *CsWOX8/AtWOX1*, *CsWOX7/AtWOX5*, *CsWOX9/AtWOX2*, *CsWOX1/OsWOX9B*, *CsWOX6/OsWOX11/12*, *CsWOX2/OsWOX9D*, *CsWOX8/OsNS1/2*, *CsWOX7/OsWOX5*, and *CsWOX9/OsWOX3* (Figure 1A).

#### 2.2.2. Gene Structure and Synteny Analysis of CsWOX Genes

To further observe the structural diversity of the *WOX* genes in sweet orange, an exon–intron diagram of the *CsWOX* genes was created with reference to their genomic and coding sequences; the number of exons varied from two to four and the number of introns from one to three in *CsWOXs* (Figure 1C). Furthermore, MCScan was used to identify duplicate gene types (Figure 1B). Almost all *CsWOX* genes were singletons. In a syntenic block (*Citrus sinensis* and *Arabidopsis*), each member belonged to the same subfamily and phylogenetic group.

### 2.3. Cis-Acting Element and Conserved Motif Analysis of CsWOX Family

#### 2.3.1. Cis-Acting Element in the Upstream Sequence of CsWOX Family

**Figure 2:**
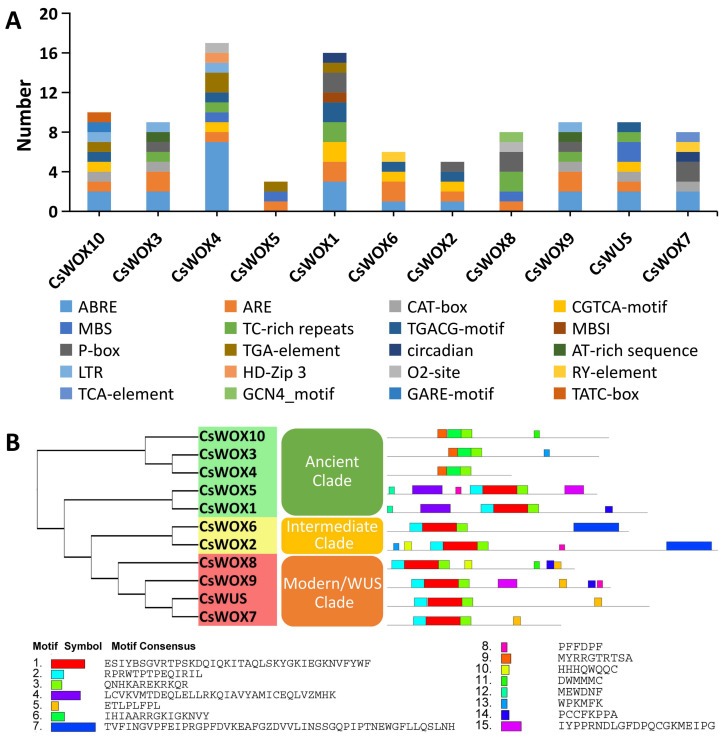
Cis-regulatory elements in promoter region of CsWOX genes and motif analysis of CsWOXs. (**A**) Cis-regulatory elements were identified in 1500 bp promoter sequence upstream of start codon of CsWOX genes using the online tool PlantCARE and PLACE. These elements are related to different functional diversity, represented by different colors. (**B**) Motif analysis of CsWOX proteins. Colors of boxes indicate different motif numbers; length of box shows motif length.

To gain further insight into the expression of sweet orange *WOX* genes, we carried out in silico analysis of potential *cis*-elements of each member of the *CsWOX* family conferring responsiveness to plant hormones (ABRE, TGA-element, P-box, CGTCA-motif, TGACG-motif, GARE-motif, TCA-element, and TATC-motif) within their promoters. *Cis*-elements related to endosperm and meristem expression (CAT-box and GCN4-motif) and biotic/abiotic stress response (TC-rich repeats, LTR, and MBS) were also found in the promoters of most *CsWOX*s (Figure 2A). *Cis*-elements involved in light responsiveness and anaerobic induction (ARE) were found in the promoter region of almost all Cs*WOX*s. Meanwhile, the *cis*-elements of whole *CsWOX* family were divided into different categories based on function prediction [50]. The result showed that the largest category of cis-elements was hormone-related, followed by stress and development-related cis-elements (Supplementary Figure S2). However, many motifs have not yet been functionally characterized and whether these motifs confer unique functional roles to *CsWOXs* remain to be further investigated.

#### 2.3.2. Motif Analysis of CsWOX Family

In addition, the conserved motifs of CsWOX proteins were analyzed by the MEME Suite. A total of 15 conserved motifs were predicted in the CsWOX proteins (Figure 2B). The size of the identified motifs ranged from 6 to 50 amino acids (Figure 2B). The results show that groups classified by phylogenetic analysis shared similar conserved motif compositions. However, there were some differences. For example, motif 3 was present in all members of the CsWOX family; motif 15 was found in just two members, CsWOX5 and CsWOX9; motif 4 was found in CsWOX5 and CsWOX1; motif 9 was found in three members, CsWOX10, CsWOX3, and CsWOX4, of the ancient clade; and motifs 7 and 5 were distinctly found in the intermediate and modern/WUS clades, respectively (Figure 2B). To some extent, these specific motifs may lead to the functional differences of *WOX* genes in sweet orange.

### 2.4. Expression of CsWOX Genes in Different Citrus sinensis Tissues and under Floral Inductive Water Deficit Conditions

**Figure 3:**
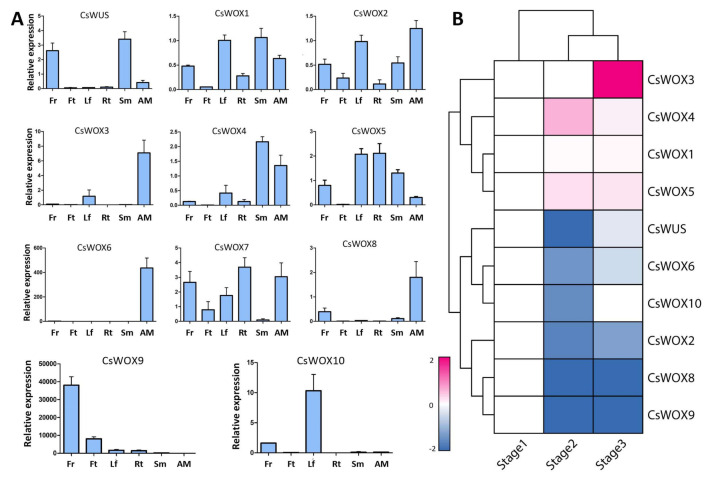
Expression of *CsWOX* genes in different *Citrus sinensis* tissues and under floral inductive water deficit conditions. (**A**) Real-time PCR validation for 11 genes expressed in different tissues: leaf (Lf), flower (Fr), fruit (Ft), stem (Sm), apical meristem (AM), and root (Rt). *CsActin* was used as internal control; mean ± SD of three biological replicates are presented. (**B**) Cluster analysis of *CsWOX*s expression based on log_2_ ratio ≥ 1. Bar denotes different expression levels and colors indicate relative signal intensities. Stage 1: one week before water deficit; stage 2: one week after beginning of water deficit; stage 3: one week after release of water deficit [51].

To further determine the function of *CsWOX* genes, their tissue-specific expression was evaluated (Figure 3A). The results revealed that the majority of *CsWOX* genes were expressed in multiple tissues, although some showed similar expression trends in different tissues. For example, *CsWUS* showed the highest expression in the flower and stem. *CsWOX1* and *CsWOX2* were constitutively expressed in all tissues, implying that they are involved in multiple developmental stages, while other *CsWOX* genes were differently expressed, suggesting that they have tissue specificity. *CsWOX4* was highly expressed in stem and apical meristem but had no expression in fruit. *CsWOX3*, *CsWOX8*, and *CsWOX6* showed similar expression patterns in apical meristem (Figure 3A). However, *CsWOX8* was expressed in the flower and *CsWOX6* had relatively high expression only in apical meristem tissue, indicating that it may be involved in apical meristem cell maintenance. *CsWOX9* was highly expressed in the flower and had low expression in the stem and leaf, indicating functional redundancy in flower development. *CsWOX10* had maximum expression in the leaf and relatively less expression in the flower. *CsWOX7* had high expression in the root. However, Cs*WOX5* had no expression in fruit (Figure 3A). Collectively, this indicates that these genes may be involved in the maintenance of stem cells and in organ development and differentiation.

To investigate the potential role of *CsWOX* genes under floral inductive water deficit conditions, we evaluated the expression patterns using previously reported RNA-seq data in lemon [51]. RNA-seq analysis was performed at stage 1, one week before the water deficit; stage 2, one week after the beginning of the water deficit; and stage 3, one week after the release of the water deficit. We categorized 10 *CsWOX* genes into three clusters and excluded the *CsWOX7* gene as it has no expression in this specific condition (Figure 3B). Cluster 1 consisted of four genes (*CsWOX3, CsWOX4, CsWOX1,* and *CsWOX5*). These genes were subsequently induced at the beginning of the water deficit condition, while *CsWOX3* had wide expression at stage 3 (Figure 3B). Such genes may be crucial for citrus flower bud differentiation. Cluster 2 genes (*CsWUS*, *CsWOX6*, and *CsWOX10*) were suppressed at stage 2 and induced at stage 3. This cluster showed upregulation in the expression of genes involved in vegetative growth after water recovery. Cluster 3 genes (*CsWOX8*, *CsWOX9*, and *CsWOX2*) were suppressed throughout the water deficit and water recovery treatment. Four genes (*CsWOX4*, *CsWOX1*, *CsWOX6*, and *CsWUS*) were differentially expressed depending on probability ≥ 0.8 and absolute value of the log_2_ ratio of ≥1 as a threshold. These findings show that *CsWOX* genes have narrow expression under water deficit conditions (Figure 3B).

### 2.5. Co-Expression Analysis of CsWOX Genes under Water Deficit Floral Initiation

**Figure 4:**
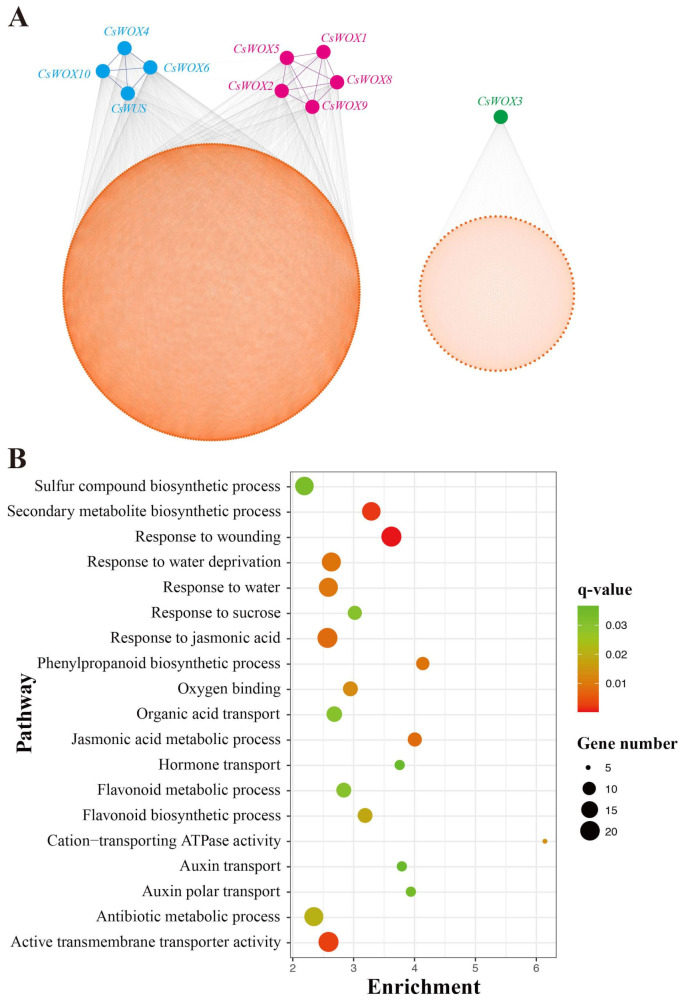
Co-expression network analysis of *CsWOXs* using data from a previous study [51]. (**A**) *CsWOXs* centered gene co-expression network under floral inductive water deficit. (**B**) Biological processes of Gene Ontology (GO) terms that were significantly augmented in *CsWOX* gene targeted network.

Co-expression analysis was done under floral inductive water deficit conditions to determine the probable role of *CsWOX* genes in citrus (Figure 4A). Co-expression analysis generally clustered 11 *CsWOX* genes into two modules, together with 1638 differential expressed genes via 42,176 interactions. For instance, module 1 was the widest co-expression group, consisting of nine *CsWOX* genes (*CsWUS*, *CsWOX8*, *CsWOX9*, *CsWOX4*, *CsWOX2*, *CsWOX5*, *CsWOX6*, *CsWOX10*, and *CsWOX1*). Genes from module 2 were co-expressed uniquely with *CsWOX3* (Figure 4A). The co-expression network and Kyoto Encyclopedia of Genes and Genomes (KEGG) analysis of *CsWOX* genes found a potential role for them in water deficit conditions and growth. The genes evaluated in the co-expression network were enriched in terms of the secondary metabolite biosynthetic process, response to wounding, water deprivation, or response to water as well as active transmembrane transporter activity (Figure 4B). The co-expression network was divided into two modules, as illustrate above, and it was important to check respective functions performed by different modules. In addition, the Gene Ontology (GO) analysis showed that these genes from various modules had diverse functions in various facets of plant development.

### 2.6. Overexpression Analysis of CsWUS in Tobacco and Gene Silencing Analysis in Lemon

**Figure 5:**
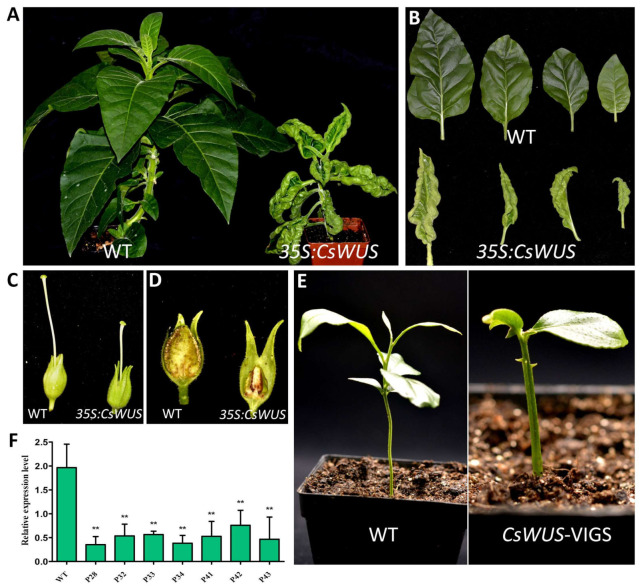
Phenotype analysis of *CsWUS* overexpression in transgenic tobacco and virus-induced gene silencing (VIGS) in lemon. (**A**) Phenotype, (**B**) leaf morphology, (**C**) flower gynoecium, and (**D**) seed pods of control and *CsWUS* transgenic tobacco. (**E**) Representative phenotypes of wild-type (WT) and *CsWUS* silencing induces thorn development in lemon. (**F**) Relative expression levels of gene silenced and control plant. *CsActin* was used as internal control; mean ± SD of three biological replicates is presented. Asterisks indicate significant differences: ** *p* < 0.01.

*CsWUS* consists of functional domains with the dual function as a suppressor or activator similar to *AtWUS*, as previously reported [5,52]. In fact, *CsWUS*, as a member of the *WUS* clade, plays an important role during the growth and development of plants [24], yet its functional role is rarely studied in citrus. Therefore, we focused on functional characterization of *CsWUS* during development and growth of citrus. Sequence alignment and phylogenetic analysis showed that Cs1g25270 has high similarity with *Arabidopsis* compared with other citrus *CsWOX* genes, and thus was named *CsWUS* (Supplementary Figure S3). To investigate the functional role of *CsWUS*, it was over-expressed in tobacco driven by the 35S promoter. A total of eight independent transgenic lines (*CsWUS-*OE) were obtained (Figure 5A). All *CsWUS* transgenic plants showed a similar phenotype (Figure 5A). *CsWUS-*OE transgenic plants had leaf lamina curled up inside, reduced leaf area, and stunted plant growth (Figure 5B). The gynoecium of the flower in *CsWUS*-OE lines was markedly smaller compared with the wild type (WT) (Figure 5C), which suggests that overexpressed *WUS* may restrict the ability of female reproductive organ development. It is worth noting that *CsWUS-*OE plants had empty seed pods with no seed development compared to WT seed pods (Figure 5D). Furthermore, the relative expression of the endogenous *NtWUS* ortholog gene was performed in *CsWUS*-OE transgenic plants, the results showed that the expression of *NtWUS* was significantly up-regulated compared with the wild-type (Supplementary Figure S4). It is known from overexpression analysis that *CsWUS* may be involved in aberrant cell division, stem cell fate, and organ development.

To further investigate the functional role of *CsWUS* in citrus, the VIGS approach was used to knock down the *CsWUS* in lemon. Transcript abundance of *CsWUS* in the positive VIGS plants, designated as TRV (tobacco rattle virus)-*CsWUS*, were repressed compared to the control seedlings (designated as TRV) that were only infiltrated with empty vector (Figure 5F). Two weeks following VIGS knockdown, conspicuous differences were noticed in plant morphology with thorn like meristem outgrowths present on the plant stems (Figure 5E). These results suggested that *CsWUS* may be involved in thorn development activity in citrus.

### 2.7. Identification of Interacting CsWUS Proteins

**Figure 6:**
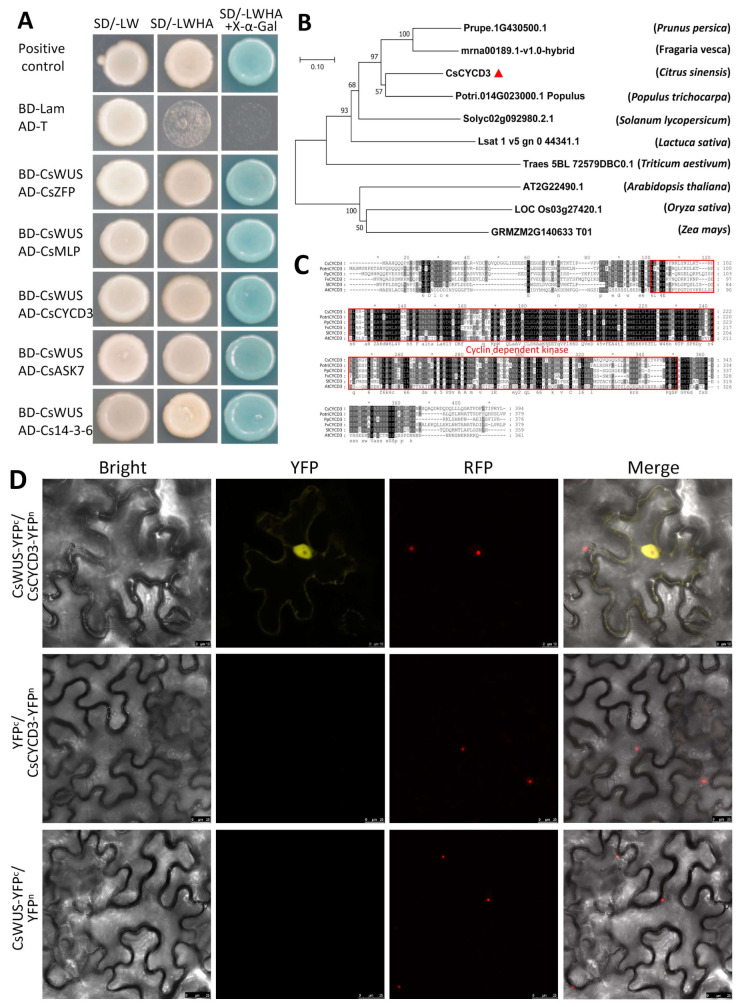
Protein interaction analysis of CsWUS protein. (**A**) Interacting identified proteins based on yeast two-hybrid screening. Yeast co-transformed with CsWUS as bait and AD-CsCYCD3, AD-Cs14-3-3, AD-CsASK7, AD-CsZFP, and AD-CsMLP as prey were dropped and deposited onto SD/-Trp-His-Ade-Leu or SD/-Trp-His-Ade-Leu medium with X-α-Gal. AD-T+BD-P53 was used as positive control and AD-T+BD-Lam as negative control. (**B**) Phylogenetic analysis of CsCYCD3 with homolog proteins. (**C**) Sequence analysis of CsCYCD3 protein with homolog proteins; CsCYCD3 from *Citrus sinensis,* AT2G22490.1 from *Arabidopsis*, Potri.014G023000.1 from *Populus trichocarpa*, mrna00189.1-v1.0-hybrid from *Fragaria vesca*, and Prupe.1G430500.1 from *Prunus persica*. (**D**) BiFC analysis in tobacco transient assays, where tobacco was co-transformed with YFPn and YFPc. Yellow fluorescent protein (YFP) images for the interaction of CsCYCD3-YFP^N^ venus with CsWUS-YFP^C^ were observed using YFP filter. The negative controls failed to yield detectable yellow fluorescence. RFP (Red mCherry) was used as a marker for nuclei. Scale bar = 20 µm.

CsWUS is generally associated with other proteins and TFs to form a transcription complex that regulates the expression of downstream genes directly or indirectly. To recognize the function of CsWUS protein, we evaluated its interaction partners via yeast two-hybrid screening. The conserved domain of CsWUS was cloned into the PGBKT7 vector. CsWUS-BD had no activation capability by the self-activation detection experiment; we used it as bait to perform yeast two-hybrid screening against the citrus mix cDNA expression libraries. Five interaction proteins were identified in at least two independent screens, indicating CsWUS interacting protein (Figure 6A).

These protein interactions are reported for the first time with CsWUS protein interaction: CsCYCD3 (Cs3g23120) ortholog of AtCYCD2;1 encodes cyclin-dependent kinase; zinc finger protein (Cs1g13130), which is an ortholog of *Arabidopsis*; miraculin-like protein (Cs7g08260) encodes soybean Kunitz super-family ortholog of AtMLP-like protein; Shaggy related protein kinase CsSK7 (Cs2g04660) ortholog of AtSK7 encodes catalytic domain of serine/threonine kinase and glycogen synthase kinase 3; and Cs14-3-3 protein (orange1.1t01991) encodes the 14-3-3 domain ubiquitous class of phosphoserine threonine binding protein. Generally, CsWUS may be related to various complexes composed of these interacting proteins.

Among these five interacting proteins, CsCYCD3 protein has a crucial role in enhancing cell division, while CsWUS protein is involved in stem cell fate. Sequence alignment and phylogenetic analysis showed that CsCYCD3 has high similarity with *Populus trichocarpa* CYCD protein compared with CYCD3 from other plants (Figure 6B). Sequence alignment showed that CsCYCD3 has high similarity with *Arabidopsis* AtCYCD2;1 (Figure 6C). Hence, it is hypothesized that CsWUS interacts with CsCYCD3 and regulates reproductive growth and stem cell fate. To further confirm the interaction between CsWUS and CsCYCD3, we performed BiFC analysis in tobacco (Figure 6D). The findings show that the interaction between CsWUS and CsCYCD3 takes place in the nucleus. Therefore, we may speculate that the interaction between CsWUS and its co-partner CsCYCD3 is involved in tissue proliferation. These results provide a basis for further evaluation of these proteins regarding undifferentiated growth in citrus.

### 2.8. Sub-Cellular Localization, CsWUS Transcription Activation Analysis and the Identification of RAP2.12 and CsHB22 Transcription Factor in Citrus sinensis

**Figure 7:**
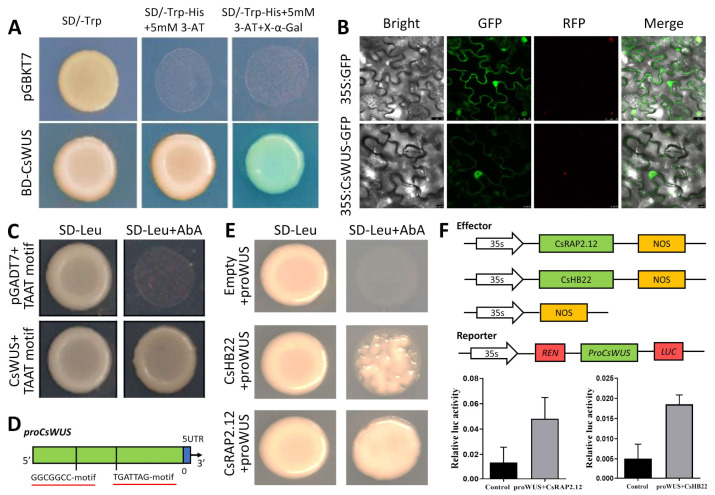
Functional analysis of *CsWUS*. (**A**)Transcriptional activation of *CsWUS*; full-length *CsWUS* fused with GAL4 DNA-binding domain then expressed in yeast strain AH109. Transformed yeast cells were dropped and deposited on selective media (SD/-Trp-His-Ade-Leu) supplemented with 3AT+x-a-gal, with negative control empty pGBKT7 vector. (**B**) Subcellular localization in tobacco cells; *GFP:CsWUS* was transiently expressed in tobacco cells under CaMV 35s promoter. Scale bar = 25 µm. (**C**) TAAT motif binding confirmation followed by yeast one-hybrid method, pGADT7 used as a negative control, TAAT+AD-CsWUS and pGADT7 was grown on SD/-Leu supplemented with Aureobasidin A [53]. (**D**) Schematic diagram of *proCsWUS* and construct of yeast one-hybrid assay. (**E**) Interacting TFs identified based on yeast one-hybrid library screening; positive transformations were determined by spotting dilution of yeast onto SD/−Leu supplemented with AbA, negative control (promoter+PGADT7). (**F**) Transient expression assay. Schematic diagram of reporter and effector constructs used for transient expression assays; co-expression of *CsRAP2.12* and *CsHB22*, *CsWUS* promoter contained GCC- and TGAT-motif.

To identify the subcellular localization of *CsWUS*, its full-length open reading frame (ORF) sequence was cloned into the pRI101 vector under 35S promoter, resulting in an in-frame fusion protein of CsWUS:GFP. The results show that the tobacco epidermal cell expressing GFPs showed cytoplasmic and nuclear staining, while CsWUS:GFP can also be detected in the whole cell, similar to a positive control (Figure 7B). To determine the transcription activity of *CsWUS*, it was fused with GAL4 DNA-binding domain (GAL4BD) and tested in yeast AH109 (Figure 7A). The result shows that *CsWUS* may act as a transcriptional activator. Consequently, the DNA binding ability of *CsWUS* gene confirms that *CsWUS* binds with (TAATTCA) motif and verifies the DNA binding ability of *CsWUS* through yeast one-hybrid (Figure 7C).

To identify TFs that regulate CsWUS, a yeast one-hybrid assay was performed using *ProCsWUS* as bait. The 2kb *CsWUS* promoter fragment was cloned and 0.5 kb core sequence (from –579 to –1003) was inserted into pAbAi and used as bait. We obtained 20 positive clones; only two genes (Cs1g16690 and Cs3g22190) were found after putative re-streaking on high stringency medium supplemented with 100 mM Aureobasidin A (Papdi, #207) [53] (Figure 7E). Cs1g16690, belonging to the AP2/ERF TF family, and Cs3g22190, belonging to the zinc finger homeodomain TF family, were identified from the citrus genome database. Meanwhile, a number of *Cis*-elements were found on *ProCsWUS*, including AP2/ERF (GGCGGCC) elements, which have been recognized by Cs1g16690. ZF-HD (TGATTAG) elements have been shown to be recognized by Cs3g22190 (Figure 7D).

The results of comparison between cDNA and genomic DNA sequences reveled that Cs3g22190 is located on chromosome 3. Alignment and phylogenetic analysis showed that this gene has high similarity with *Arabidopsis* ZF-HD homeobox protein 22 (At4g24660), and thus was named *CsHB22* (Supplementary Figure S5). It is composed of a 630 bp full-length ORF encoding a 209 amino acid putative protein. The *CsHB22* consists of ZF-HD dimer protein and homeobox domain, consistent with previous reports on *HB22* protein. A comparison was made between Cs1g16690 cDNA and genomic DNA located on chromosome 1. Alignment and phylogenetic analysis showed that this gene has similarity with *Arabidopsis BRELATED TO AP2.12* (*AT1G53910*) and it was named *CsRAP2.12* (Supplementary Figure S6). It is composed of 1170 bp, ORF encoding 326 amino acids. CsRAP2.12 consists of AP2-ERF domain and ORC2 super-family.

To further confirm *CsRAP2.12* and *CsHB22* binding to the *CsWUS* promoter, we investigated whether *CsRAP2.12* and *CsHB22* activated or suppressed *ProCsWUS* in vivo by performing dual luciferase assay on tobacco leaves. In this study, *CsRAP2.12* and *CsHB22* were used as effectors and two constructs consisting of *ProCsWUS* were used as reporters (Figure 7F). The results show that co-transformation of effectors and reporters significantly elevated the promoter activity of *CsWUS* (Figure 7F). Taken together, this suggests that these two TFs may be involved in phenotypic complementation of *CsWUS* and alter growth compared with the control by regulating the intrinsic hormonal and developmental pathways.

### 2.9. Expression of CsRAP2.12, CsHB22, and CsCYCD3 in Different Citrus Sinensis Tissues and under Floral Inductive Water Deficit Conditions

**Figure 8:**
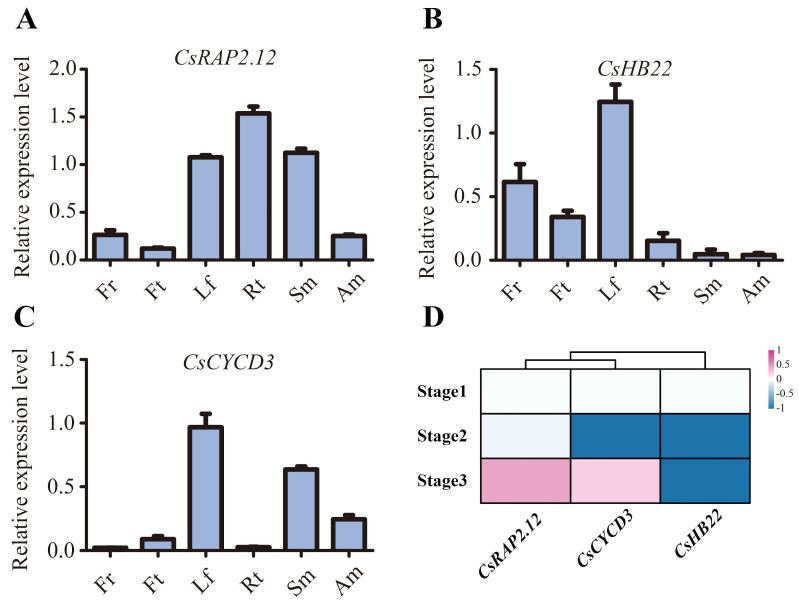
Expression of *CsRAP2.12*, *CsHB22*, and *CsCYCD3* in different *Citrus sinensis* tissues and under floral inductive water deficit conditions (**A**–**C**) Real time PCR investigation for *CsRAP2.12* (**A**), *CsHB22* (**B**), and *CsCYCD3* (**C**) expressed in different tissues of sweet orange: leaf (Lf), flower (Fr), fruit (Ft), stem (Sm), apical meristem (Am), and root (Rt). *CsActin* was used as internal control; Values are the means ± SE of at least three replications for the relative expression. (**D**) Cluster analysis of *CsRAP2.12*, *CsHB22* and *CsCYCD3* expression based on log_2_ ratio ≥ 1. Bar denotes different expression levels and colors indicate relative signal intensities. Stage 1: one week before water deficit; stage 2: one week after beginning of water deficit; stage 3: one week after release of water deficit [51].

To further investigate the spatial expression pattern of *CsRAP2.12*, *CsHB22*, and *CsCYCD3*, their tissue specific expression was evaluated in different *Citrus sinensis* tissues including the leaf, flower, fruit, stem, apical meristem, and root (Figure 8A–C). The results showed that *CsRAP2.12* showed a higher level of expression in roots, leaves, and stems, and a lower level of expression in flowers, fruits, and apical meristem (Figure 8A). *CsHB22* was relatively highly expressed in the leaf, flower, and fruit (Figure 8B). Moreover, we also performed tissue specific expression analysis of *CsCYCD3* (Figure 8C). *CsCYCD3* was mainly expressed in the leaf, stem, and apical meristem (Figure 8C). It is worth noting that all three genes showed high levels of expression in the leaves. Overall, these three genes presented a very broad expression pattern, implying that they may play multiple roles in the growth and development of sweet oranges.

To explore the potential role of *CsRAP2.12*, *CsHB22*, and *CsCYCD3* under floral inductive water deficit conditions, we also evaluated their expression patterns using previously reported RNA-seq data in lemon [51]. The result showed *CsRAP2.12* was highly expressed after water recovery while *CsHB22* is not induced under floral inductive water deficit conditions (Figure 8D). In addition, *CsCYCD3* was more highly expressed at stage 3 than stage 2 (Figure 8D). These results suggest that they may also play an important role under floral inductive water deficit conditions.

## 3. Discussion

The *WOX* family plays a crucial role in shoot apical meristem and embryonic development, stem cell activity, and various other developmental processes in plants [5,22,44]. Due to the significance of their functions, research on plants has become more urgent and widespread. Previous genome-wide analysis of the *WOX* gene family was done in some important plant species [13,18,44,54]. However, there is no report of genome-wide analysis of the *WOX* family in citrus. In this study, we identified 11 putative *CsWOX* genes in the citrus reference genome. Based on their phylogenetic relation with *Arabidopsis* and rice WOX proteins, CsWOX proteins were split into three clades (modern/WUS, intermediate, and ancient), consistent with already reported classifications in different plant species [21,24,55]. However, synteny between *CsWOX* genes and their *Arabidopsis* homologs was less than expected.

Identifying the role of *CsWOX* genes by tissue-specific expression and co-expression analysis is an important and useful tool. For example, *CsWOX1* a putative ortholog of *AtWOX13* has highly expressed in the flower, root, and apical meristem. The *WOX13* mutant exhibited slightly wider fruits with a reduced number of lateral roots in *Arabidopsis* [9]. However, *GhWOX13* in cotton is involved in hormonal mediation of fiber elongation [56]. In this study, tissue-specific expression analysis suggests that *CsWOX1* performs a similar function to *AtWOX13* [9]. *CsWOX7* is a putative ortholog to *AtWOX5* in *Arabidopsis*, and *CsWOX5* was shown to be involved in root meristem maintenance [12]. Further studies of *AtWOX5* orthologs in maize (*ZmWOX5*), poplar (*PtoWOX5a*), and rice (*OsWOX5*) showed a similar conserved function and molecular mechanism in root development [57,58]. Tissue-specific expression analysis showed that *CsWOX7* is highly expressed in root tissue, indicating that it may be involved in root development of sweet orange. *CsWUS* was found as a putative ortholog of *AtWUS* and *OsWUS*. *WUS* is a critical regulator and encodes a homeodomain protein that is mandatory for stem cell activity [59]. Nevertheless, *AtWUS* regulates maintenance of stem cells in floral meristems, and its expression level influences the number of flower organs that develop [60]. *AtWUS* is a bi-functional TF that acts to repress stem cell regulation and activate floral patterning [42]. Previous findings explained the conserved functioning of *WUS* genes in *Gossypium hirsutum*, *Arabidopsis*, *Medicago truncatula*, *Glycine max*, *Triticum aestivum*, *Coffea canephora*, *Nicotiana tabacum*, *Oryza sativa*, and *Chrysanthemum morifolium* [5,13,31,39,40,44,61,62]. High expression of *CsWUS* in the flower and stem suggests that it may be linked with maintenance of stem cell and flower organ development.

Interestingly, CsWUS-OE in tobacco exhibited a developmental role and induced ectopic growth. Overexpressed plants had malformed leaves, stunted growth with limited gynoecium development, and no seed development. Similar results were observed with *AtWUS* overexpression in tobacco [40]. *CsWUS* consists of functional domains considering its dual function as an activator similar to *AtWUS*, as previously reported [5,52]. Results show that the *WUS* gene has a conserved developmental role in *Arabidopsis* and sweet orange. However, gene silencing of *CsWUS* revealed conserved functioning in stem cell activity involved in thorn development in lemon.

To further investigate the regulation mechanism of *CsWUS*, we performed a yeast two-hybrid analysis using CsWUS as a bait, and five interacting protein partners of CsWUS were identified. Phenotypic functional complementation experiment with *CsWUS* confirmed involvement in aberrant cell division and stem cell activity. *CsCYCD3* ortholog in *Arabidopsis* enhances cell division and plays a vital role in tissue proliferation, such as in the meristem and young leaves [9,63]. Cyclins are well conserved in functioning, and therefore have been comprehensively recognized in plants. Previous studies reported that *CYCD3* defines distinct developmental zones and is locally regulated by *CYCLOIDEA* [64]. In addition, *CYCLOIDEA* (*CYC2-like*) and *WUS* regulate reproductive organ development in *Chrysanthemum morifolium* [44]. Furthermore, we found physical interaction between CsCYCD3 and CsWUS in the tobacco nucleus. Therefore, we may predict that CsCYCD3 and CsWUS complex might be involved in stem cell activity and promote ectopic growth in citrus. The conserved mechanism of CsCYCD3 and CsWUS in citrus needs to be studied further. Subsequently, other interacting proteins, such as 14-3-3 protein 6, shaggy related protein kinase, zinc finger homeodomain, and MLP-like protein are involved in various developmental, hormonal signaling, and stress responses in *Arabidopsis* [65,66,67,68]. These proteins’ triggering mechanisms still need to be further elucidated.

In addition, we identified two TFs (*CsRAP2.12* and *CsHB22*) binding to the *CsWUS* promoter by yeast one-hybrid library screening. These results indicate that the expression of *CsRAP2.12* and *CsHB22* activates the expression of *CsWUS* by binding to its promoter. *CsRAP2.12* is an ortholog of *AtRAP2.12* and belongs to the ERF-VII TF family. *AtRAP2.12* and its orthologs shared a conserved AP2 domain that was mandatory for protein–DNA interaction. *AtRAP2.12* involved in hypoxia tolerance and reduced growth in the presence of oxygen in *Arabidopsis* [53]. The ubiquitin-dependent N-end rule pathway for protein degradation, functions as an oxygen sensing mechanism in *Arabidopsis* [69]. Furthermore, the presence of molecular oxygen affected the stability of ERF-VII proteins, implying that the role of oxygen sensing was mediated via the N-end rule protein degradation pathway [70]. The N-end rule pathway, in particular, stagnates the stress response in plants, enabling optimal growth and development. Meanwhile, *RAP2.12* was also an ethylene responsive TF, as ethylene is known to influence the growth and development of leaves and can be independent of or dependent on its interaction with other hormones [71,72]. We observed malformed curled leaves in CsWUS-OE lines, which showed similar phenotype behavior to those plants grown in the presence of polar auxin transport inhibitors. It is known that auxin regulates the cell division phase during leaf expansion [40]. We may speculate that the response of leaf growth to ethylene is likely to be auxin-dependent or auxin-independent [33]. In addition, overexpression of stabilized *RAP2.12* alters the leaf phenotype by regulating non-hypoxic target genes in *Arabidopsis* [73]. Therefore, we suggest that *CsRAP2.12* binds with promoter of *CsWUS*, and this interaction may be involved in hormonal signaling that regulates leaf development.

ZF-HD belongs to a subfamily of homeodomain TFs that has not been well characterized functionally in plants [68]. In *Arabidopsis*, 14 members of the ZF-HD family have been identified and predominantly expressed in flower tissues [74]. The *CsHB22* ortholog in *Arabidopsis* (AT4G24660) was highly expressed in flower tissues [68,74]. However, its specific function needs further investigation in *Arabidopsis*. Recently, a member from the ZF-HD family (*OsZHD2*) was described that promotes root and meristem activity by biosynthesis of ethylene in rice [75]. Besides the regulatory role in the growth and development, some *ZF-HD* genes may play a vital role in response to abiotic stresses such as drought, heat, cold, and salt [76]. Recent studies indicate that ZF-HD is induced by cold stress, NaCl, and PEG in wheat [77]. Consistent with all previous reports, we also found the flower gynoecium in CsWUS-OE was smaller than that in WT. Thus, overexpression of *CsWUS* gene in sweet orange is expected to retard the ability to develop female gametophytes. In addition, tissue specific expression showed that *CsRAP2.12*, *CsHB22*, and *CsCYCD3* were expressed in the flower, leaf, stem, and apical meristem. Taken together, we therefore hypothesized that the interaction of CsWUS-CsCYCD3, *ProCsWUS-CsRAP2.12*, and *ProCsWUS-CsHB22* may be involved in development of sweet orange leaf, flower, stem, and apical meristem. However, further studies are required to confirm these initial findings.

## 4. Materials and Methods

### 4.1. Plant Materials

Tissues samples of leaf, flower, root, apical meristem, stem, and young fruit were obtained from sweet orange grown at the National Citrus Research Breeding Center, Huazhong Agriculture University, Wuhan, China. Flower and fruit samples were collected in flowering and fruiting seasons, respectively. The samples were collected for *CsWOX* expression analysis and promptly placed in liquid nitrogen and further preserved at –80 °C for RNA extraction and expression analysis.

### 4.2. Identification of WOX Genes in Sweet Orange

To recognize all putative *WOX* genes of sweet orange, a local BLAST search using the citrus database (http://citrus.hzau.edu.cn/orange/) was done [48]. Two BLASTP approaches were implemented to search the sweet orange *WOX* genes. First, 15 known *Arabidopsis WOX* genes were downloaded from the *Arabidopsis* Information Resource (TAIR) database and used to query the citrus database, and candidate genes were recognized by BLASTP search scores of ≥100 and e-value of ≤e^−10^. Second, the same procedure was done using all known rice genes downloaded from the NCBI database, which were used as query sequences [78]. The 11 identified *WOX* genes were designated as *CsWOX* genes.

### 4.3. Phylogenetic Analysis and Gene Structure of CsWOX Family

To explore the phylogenetic relationship between sweet orange, *Arabidopsis*, and rice, a phylogenetic tree was constructed using the Clustal Omega program (Guide Tree) (www.ebi.ac.uk/Tools/msa/clustalo/) follow the default parameters. The corresponding phylogenetic tree data was downloaded, and the phylogenetic tree was drawn using Interactive Tree of Life (IToL) v. 4 (https://itol.embl.de/), scale bars correspond to 0.1 substitution [79,80]. In this study, *Arabidopsis* and rice *CsWOX* proteins were used as the out group. The protein sequences of *Arabidopsis* and rice were downloaded from the TAIR and NCBI databases, respectively. Gene Structure Display Server 2.0 was used to evaluate the exon/intron structures of *CsWOX* genes [81].

### 4.4. Analysis of Conserved Motif and Predicted Subcellular Localization

To study the structural modification of *CsWOX* genes, the conserved motifs in the encoded CsWOX proteins were analyzed by Multiple Expectation Maximization for Motif Elicitation (MEME) v. 5.3 (https://meme-suite.org/meme/tools/meme) with default parameters [50]. Conserved motifs were identified with the motif widths of 6–50 residues. The online program WOLF PSORT II (http://www.genescript.com/wolfpsort.html) was used to predicate subcellular positions of *CsWOX* genes with default parameters, the organism type selected plants [82].

### 4.5. Analysis of Protein Structures

The biochemical properties of CsWOX proteins such as amino acid composition, molecular weight (MW), theoretical pI, instability index, aliphatic index, and grand average of hydropathicity (GRAVY) were obtained by an online tool on the Bioinformatics Resource Portal ExPASy server (http://web.expasy.org/protparam/) with default parameters [83]. The protein structure of CsWOX proteins was predicted by the SWISS-MODEL (https://swissmodel.expasy.org) online programs with default parameters [82].

### 4.6. Analysis of Cis-Regulatory Elements, Chromosomal Location, and Synteny

To examine the possible regulatory mechanisms of *CsWOX* genes, a 1.5 kp upstream promoter region from the start codon of each gene was taken from the sweet orange genome database (http://citrus.hzau.edu.cn/orange/) [48]. The cis-elements of *CsWOX* promoter were identified using the PLACE (https://www.dna.affrc.go.jp/PLACE/?action=newplace) and PlantCARE programs (http://bioinformatics.psb.ugent.be/webtools/plantcare/html/) [50,84]. The gene loci for *WOX* genes were also downloaded from the sweet orange genome database [48]. The MCScanX toolkit was used for synteny detection [85]. A synteny diagram was created using Circos software version 0.63 [86].

### 4.7. Expression Profile of CsWOX Genes in Sweet Orange

Spatial expression of *CsWOX* genes in various organs was analyzed by qRT-PCR. Total RNA was isolated from young fruits, flowers, healthy leaves, roots, stems, and apical meristems of sweet orange. cDNA synthesis was conducted with a PrimeScript^®^ RT reagent kit (Takara, Dalian, China) following the manufacturer’s instructions. The synthesized cDNA was diluted 1:10 as a template for qRT-PCR. The primers used are listed in Supplementary Table S1. Real-time PCR was performed with Hieff^®^ qPCR SYBR^®^ Green Master Mix (Yeasen Biotech Co., Ltd., Shanghai, China) on an ABI PRISM 7000 system (Applied Biosystems) using 1 µL of cDNA template, 8 µL of double distilled water, and 0.5 µL of forward and reverse primers (10 µM) in the following PCR condition: 95 °C for 5 min, 95 °C for 10 s, 55–60 °C for 20 s, 72 °C for 20 s, and 40 cycles. The relative expression level of target genes was calculated using the 2^−∆∆Ct^ method by normalizing *CsActin* as described previously [87].

### 4.8. Co-Expression Evaluation of CsWOX Genes during Induction of Floral Water Deficit

The specific role of *CsWOX* genes was evaluated through published transcriptome data on lemon bud under floral inductive water deficit conditions [51]. Co-expression network of *CsWOX* genes was created, and the relationships between two genes above 0.85 were retained and then visualized as a comparison in Cytoscape. Co-expression gene networks were evaluated through Gene Ontology (GO) and Kyoto Encyclopedia of Genes and Genomes (KEGG) using the clusterProfiler and pathview packages in R, as defined in [88].

### 4.9. Subcellular Localization, Motif Binding, and Transactivation Activity Assay of CsWUS

The coding sequence of *CsWUS* without stop codon was fused into PRI101 vector containing *GFP* gene under control of CaMV 35S promoter to form *35Ss:CsWUS-GFP*. The *35S:CsWUS-GFP* and *35S:GFP* constructs were transformed into *Agrobacterium tumefaciens* GV3101. The 35S:CsWUS-GFP and *35S:GFP* constructs were transiently expressed in *Nicotiana* *benthamiana* leaf epidermal cells. After 36 h of incubation, leaf cells were examined by confocal microscopy as described previously [89]. The full-length coding sequence of *CsWUS* was inserted into pGBKT7 as bait according to the manufacturer’s instructions. The bait clone with the empty prey pGADT7 and co-transformed cells on SD/-Ade/-His/-Leu/-Trp medium was transformed into yeast AH109 strain. Transactivation activity was determined by a previously reported method [19]. Cs*WUS* DNA binding activity was investigated by the Matchmaker^TM^ Gold Yeast-one Hybrid Library screening system according to the user manual (Clontech, Mountain View, CA, USA). Three tandem repeats of the predicted TAATTCA motif were inserted into the pAbAi vector and transformed in the yeast-one hybrid system according to the manufacturer’s instruction. The yeast clones expressing pAbAi and pGADT7-CsWUS were grown normally on SD/-Leu medium with AbA ng/mL [90]. The experiment was repeated three times. The primers used are listed in Supplementary Table S2.

### 4.10. Construct p35s-CsWUS Preparation and Overexpression in Tobacco Plants

The full-length coding sequence of *CsWUS* was used for overexpression analysis. For this analysis, CDS of *CsWUS* was cloned into pBI121 vector by replacing *GUS* gene (Supplementary Table S1). The sequence was then inserted into the pBI121 vector. The vector was transformed into *Agrobacterium tumefaciens* GV3101 by the heat shock method. A previously described method of *Agrobacterium* mediated transformation was used in tobacco [91]. The transgenic plants T_0_ were confirmed by PCR amplification.

### 4.11. Vector Construction and VIGS of CsWUS in Lemon

The tobacco rattle virus (TRV) system (pTRV-RNA1 and pTRV-RNA2) was used for VIGS analysis. A 428 bp gene fragment of *CsWUS* was cloned into the pTRV-RNA2 vector to produce the pTRV2: *CsWUS* construct. pTRV1, TRV2 (negative control), and pTRV2-CsWUS were transformed into *Agrobacterium tumefaciens* strain GV3101 [89]. Agro-infiltration proceeded by dipping germinating lemon seeds with a shoot length of around 1 cm in a bacterial suspension in a vacuum chamber. The plants were dried with filter paper after vacuum infiltration and grown in the dark for 3 days, then sown in soil containers under a growth chamber at 25 °C, 16 h light/8 h dark [89]. After 2 weeks, DNA of each seedling was extracted with a DNeasy Plant Mini kit (Qiagen, Hilden, Germany) and subjected to genomic PCR using one pair of primers for detection of positive plants (Table S1), and qRT-PCR was done for each positive plant to measure the transcript level of *CsWUS* [92]. The primers used are listed in Supplementary Table S1.

### 4.12. Yeast Two-Hybrid Screening

The yeast two-hybrid library screening was completed with the Matchmaker Gold Yeast Two-Hybrid system (Takara Bio, Beijing, China), using yeast strain AH109. Conserved domain of *CsWUS* was inserted into pGBKT7 vector and used as bait. Single yeast clones were selected on solid high-stringency SD/-Ade/-His/-Leu/-Trp medium and grown on liquid SD/-His/-Leu medium. Recombinant pGADT7 plasmids with cDNA inserts were selected, re-transformed, and checked by X-α-Gal filter-lift assay before sequencing. The selected query was reconfirmed through NCBI, phytozome, and citrus genome databases for further identification of corresponding genes. After library screening, these recombinant plasmids were transformed again on yeast high-stringency SD/-Ade/-His/-Leu/-Trp X-α-Gal medium [13]. The primers used are listed in Supplementary Table S1.

### 4.13. Bimolecular Florescence Complementation Assay (BiFC)

To investigate the bimolecular florescence complementation of CsWUS and interacting proteins, their ORF sequences without stop codon were amplified and cloned into pUC-SPYNE (nYFP) and pUC-SPYCE (cYFP) vectors. Full-length CDS of *CsCYCD3* was inserted into pFGC-nYFP vector to generate *N*-terminal in-frame fusions with *N*-YFP, while *CsWUS* coding sequences were cloned into pFGC-cYFP vector to form *C*-terminal in-frame fusions with C-YFP. All plasmids were transformed into *Agraobacterium* GV3101, and infiltration of tobacco leaves was performed following a previously described method [13]. After 36 h, tobacco leaves were observed under a Leica confocal laser scanning electron microscope.

### 4.14. Yeast One-Hybrid Screen and Assay

The 0.5 kb *CsWUS* promoter was amplified and cloned into pAbAi and used as bait, and pGADT7 library was used as a prey. Yeast one-hybrid library screening assay was performed using the Matchmaker^TM^ Gold Yeast-one Hybrid Library system (Clontech, Mountain View, CA, USA) according to the manufacturer’s instructions. Protein-DNA interaction was revealed by the growth ability of co-transformed yeast cells on high–stringency SD/-leu medium supplemented with AbA following the manufacturer’s protocol [19].

### 4.15. Dual Luciferase Reporter Assay

The CDS of *CsHB22* and *CsRAP2.12* was cloned into pGreenII 62-SK vector using the ClonExpressTM II One Step Cloning Kit (Vazyme Biotech Co., Ltd., Nanjing, China), and this construct was utilized as effector plasmid. The *CsWUS* promoter with specific binding motifs was cloned and inserted into pGreen0800-LUC vector plasmid to attain the reporter plasmid. The empty pGreenII 62-SK vector was used as control effector and *CsRAP2.12* and *CsHB22* were used as treatment effectors. *Agrobacterium* pSoup-19 was used for transformation of the effectors and promoters. For transient gene expression analysis, the reporter and effector recombinant plasmid constructs were co-transformed into leaves of *Nicotiana benthamiana*. After 2–3 days of infiltration, a Dual-Luciferase^®^ Reporter Assay System (Promega Biotech Co., Ltd., Beijing, China) was utilized to qualify LUC and REN activity according to the manufacturer’s instructions. At least 6 biological replicates were organized for each co-transformation.

## 5. Conclusions

This study provides a genomic framework for the citrus *WOX* gene family and its phylogenetic relation with rice and *Arabidopsis*. A total of 11 *CsWOX* genes were identified from the citrus genome. Bioinformatics analysis, including gene structure, conserved motif, *cis*-regulatory elements, protein physiochemical properties, predicted structure, and subcellular localization was performed, providing a framework for further study of this gene family in citrus. Comprehensive analysis of spatial expression patterns and co-expression analysis suggest that the *WOX* gene family is probably involved in distinct developmental mechanisms and the response to water deficit conditions in citrus. Functional analysis of *CsWUS* shows that *CsRAP2.12* and *CsHB22* regulate *CsWUS* expression. Further, CsWUS was found to interact with CsCYCD3. Ectopic overexpression of *CsWUS* is involved in aberrant cell development, malformed leaves, and defective gynoecium and ovary development. *CsWUS* gene silencing displayed radially symmetric thorn-like outgrowth in lemon. Thus, we assumed that *CsRAP2.12* and *CsHB22* regulates *CsWUS* expression, which further interacts with CsCYCD3, which may be involved in stem cell activity and other intrinsic development in citrus. The functional characterization of *CsWUS* genes the necessary foundation for follow-up more research to analyze the role of *CsWOX* genes in citrus development.

## Figures and Tables

**Table 1:** Characteristics of *Citrus sinensis WOX* genes.

Name	Genome ID	Chromosome	Start Site	End Site	CDS bp	Protein Length (aa)
*CsWUS*	Cs1g25270	Chr1	27428553	27430267	876	291
*CsWOX1*	Cs1g26550	Chr1	28546972	28548579	840	289
*CsWOX2*	Cs2g05310	Chr2	2845364	2848185	1104	367
*CsWOX3*	Cs2g16790	Chr2	13618391	13620633	1011	336
*CsWOX4*	Cs3g23280	Chr3	25600272	25601376	654	217
*CsWOX5*	Cs3g27390	Chr3	28411631	28414854	701	233
*CsWOX6*	Cs5g27430	Chr5	30010095	30011889	807	268
*CsWOX7*	Cs7g31470	Chr7	31300824	31301506	582	193
*CsWOX8*	Cs8g17610	Chr8	20428816	20430708	627	208
*CsWOX9*	Cs8g18280	Chr8	20929316	20930953	747	248
*CsWOX10*	orange1.1t00075	ChrUn	1445775	1448402	1059	352

CDS = coding sequence; Chr = chromosome; aa = amino acid; Un = unknown.

**Table 2:** Protein composition and physiochemical characteristics of CsWOX proteins.

Name	GRAVY	Aliphatic Index	Major Amino Acids Content [49]	Predicted Localization	Instability Index	MW (Da)	pI
CsWUS	–0.958	44.95	S (14%), G (11%), N (7.9%)	nucl	47.95	31,866.59	6.66
CsWOX1	–0.731	70.61	Q (8.6%), S (8.2), L (8.6%)	nucl, chlo	47	30,792.23	6.26
CsWOX2	–0.52	70.87	Q (8.4%), S (12%), P (7.9%)	chlo, nucl, cyto_nucl, mito	62.15	40,473.19	6.76
CsWOX3	–1.359	39.49	N (14.0%), S (13%), T (11%)	Nucl	46.22	26,406.6	10.21
CsWOX4	–1.136	37.54	S (13%), T (10%), R (13.8%)	nucl, mito, cyto_nucl, extr	52.15	15,437.5	11.5
CsWOX5	–0.811	59.44	Q (10.3%), S (6%), A (6%)	nucl	52.52	26,715.1	5.46
CsWOX6	–0.371	67.31	S (11.9%), G (8.2%), A (7.5%)	nucl, chlo, cyto	61.21	29,123.41	5.61
CsWOX7	–0.927	59.53	S (7.8%), T (6.7%), G (6.2%)	nucl, chlo, cyto	50.27	22,461.97	6.25
CsWOX8	–0.756	60.48	Q (11.1%), S (7.2%), L (8.7%)	nucl, cyto, mito, plas	68.12	24,103.7	9.33
CsWOX9	–0.775	55.48	G (8.5%), S (7.3%), Q (7.3%)	nucl, cyto, extr	59.76	27,672.89	6.3
CsWOX10	–0.958	50.85	S (10.2%), N (13%), G (9.3%)	nucl, cyto	36.56	26,748.22	10.03

MW: molecular weight; pI: isoelectric point; GRAVY: grand average of hydropathicity; G: glycine; L: leucine; N: asparagine; P: proline; Q: glutamine; R: arginine; S: serine; T: threonine; extr: extracellular; chlo: chloroplast; cyto: cytoplasm; mito: mitochondria; nucl: nucleus; plas: plastid.

## Data Availability

Not applicable.

## Supplementary Materials

The following are available online at https://www.mdpi.com/article/10.3390/ijms22094919/s1, Figure S1: Predicted 3D-structure of CsWOX proteins, Figure S2: The distribution of identified motif of whole *CsWOX* family based on their biological functions. Figure S3: Phylogenetic and sequence alignment of CsWUS with its homologs. (A) Phylogenetic tree of CsWUS with its homolog proteins. (B) Sequence analysis of CsWUS protein with its homolog proteins. CsWUS from *Citrus sinensis*; LOC Os04g56780 from *Oryza sativa* (OsWUS); Bradi5g25113.1.p from *Brachypodium distachyon* (BradiWUS); Cc07g10660 from *Coffea canephora* (CcWUS); AT2G17950 from *Arabidopsis* (AtWUS); XP007203178.1 from *Prunus persica* (PpWUS); TraesCS2A02G491900 from *Triticum aestivum* (TaWUS); KAF7004049 from *Triticum aestivum* (TaWUS); Potri.005G114700.1 from *Populus trichocarpa* (PotriWUS1). Bold red line indicates conserved domain of these proteins, Figure S4: (A) Phenotypic analysis of some transgenic lines from *CsWUS*. (B) Relative expression of endogenous *WUS* homolog (*NtWUS*) in transgenic *CsWUS*-OE tobacco lines. *NtActin* was worked as internal control; the mean ± SD of three biological replicates is presented, Figure S5: Phylogenetic and sequence alignment of CsHB22 with its homologs. (A) Phylogenetic tree of CsHB22 with its homolog proteins. (B) Sequence analysis of CsHB22 protein with its homolog proteins. CsHB22 from *Citrus sinensis*; LOC_Os09g29130.1 from *Oryza sativa* (LOC_Os09g2); XP_016510094.1 from *Nicotiana tabacum* (XP_0165100); Glyma.20G214300.1.p from *Glycine max* (Glyma.20G); AT4G24660.1 from *Arabidopsis* (AT4G24660); Potri.013G108900.1 from *Populus trichocarpa* (Potri.013G). Bold red line indicates conserved domain of these proteins, Figure S6: Phylogenetic and sequence alignment of CsRAP2.12 with its homologs. (A) Phylogenetic tree of CsRAP2.12 with its homolog proteins. (B) Sequence analysis of CsRAP2.12 protein with its homolog proteins. CsRAP2.12 from *Citrus sinensis*; Csa19g0208 from Camelina sativa (Csa19g020890.1); AT1G53910 from *Arabidopsis* (AT1G53910.1); Potri.003G from *Populus trichocarpa* (Potri.003G071700.1); Solyc03g12 from *Solanum lycopersicum* (Solyc03g123500.2.1); Traes_5BL from *Triticum aestivum* (Traes_5BL_7F84602F3.1). Bold red line indicates conserved domain of these proteins, Table S1: Primers used in this study.
