# Supplementary material for: Genome-Wide Identification and Expression Profiling of the WOX Gene Family in Citrus sinensis and Functional Analysis of a CsWUS Member
Source: Int J Mol Sci. 2021 May 6;22(9):4919. doi: 10.3390/ijms22094919 (PMC8124563; doi:10.3390/ijms22094919)
Supplement: Supplementary file 1 [file ijms-22-04919-s001.zip › ijms-1204466-supplementary.pdf]

International Journal of
Molecular Sciences

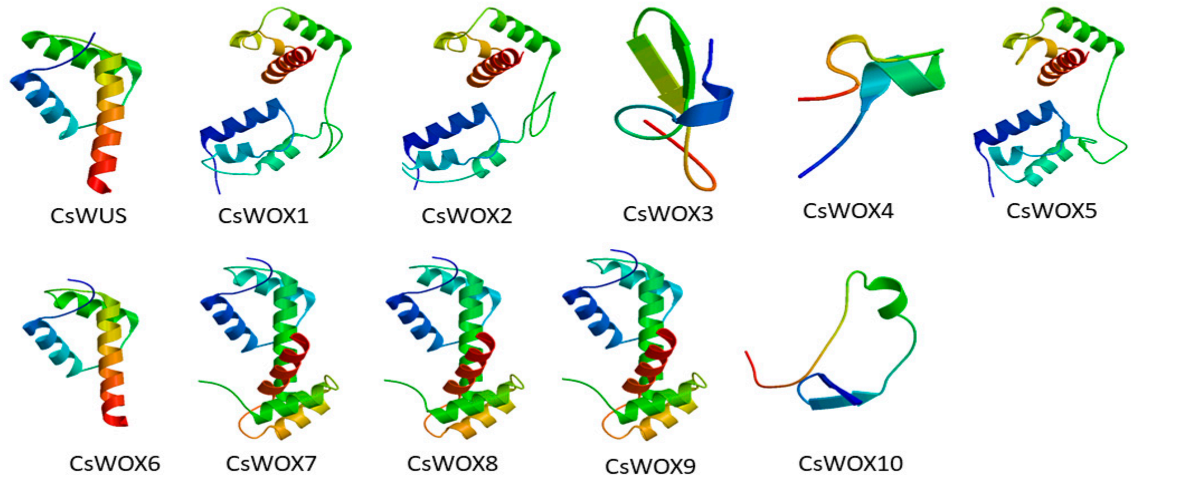

Figure S1. Predicted 3D-structure of CsWOX proteins.

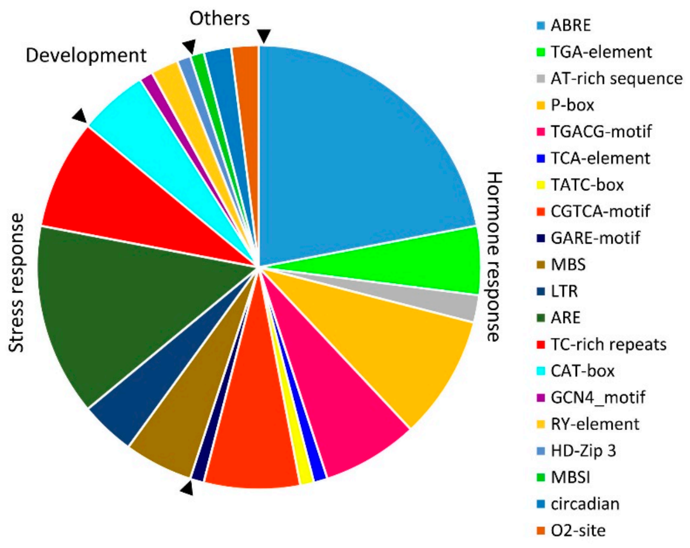

Figure S2. The distribution of identified motif of whole CsWOX family based on their biological functions by FIMO tool.

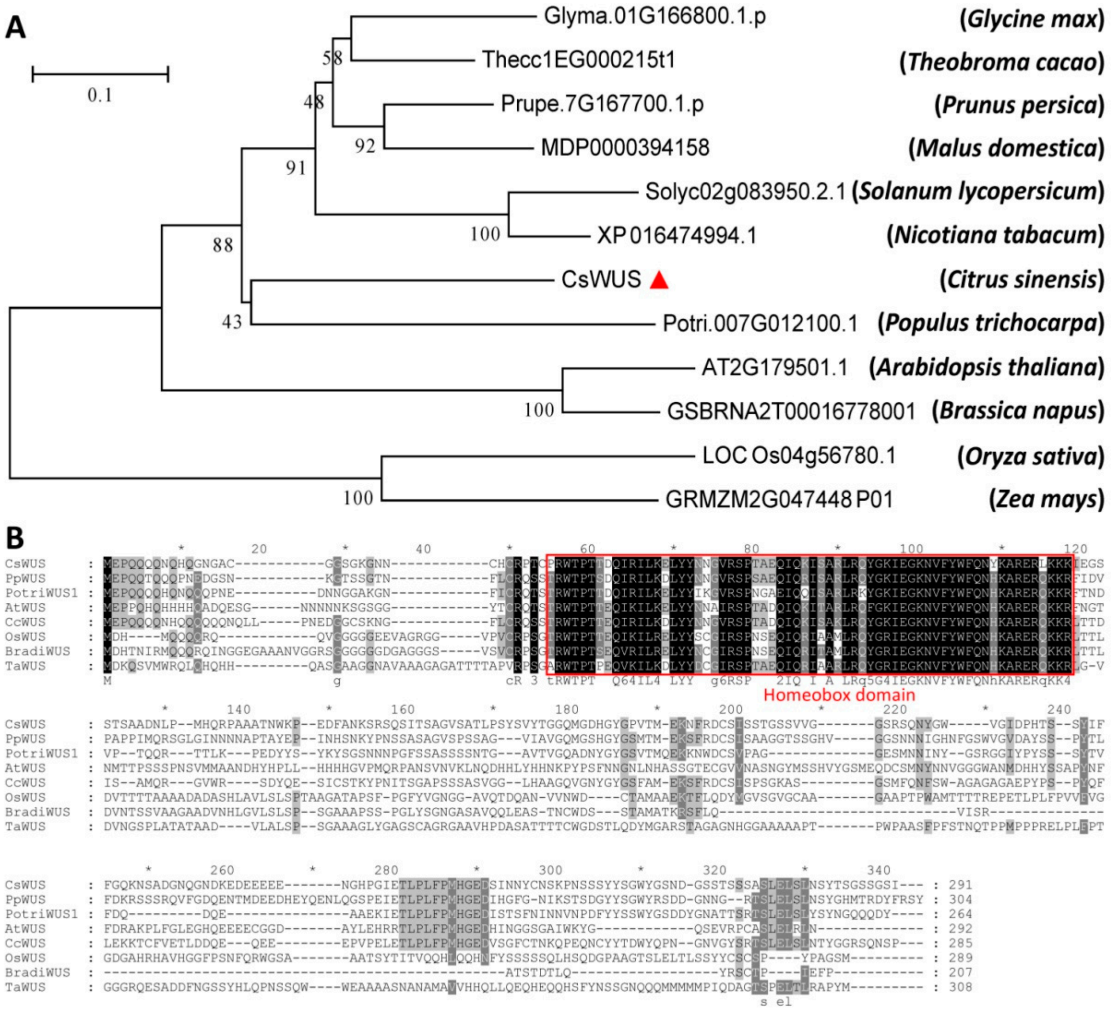

Figure S3. Phylogenetic and sequence alignment of CsWUS with its homologs. (**A**) Phylogenetic tree of CsWUS with its homolog proteins. (**B**) Sequence analysis of CsWUS protein with its homolog proteins. CsWUS from *Citrus sinensis*; LOC Os04g56780 from *Oryza sativa* (OsWUS); Bradi5g25113.1.p from *Brachypodium distachyon* (BradiWUS); Cc07g10660 from *Coffea canephora* (CcWUS); AT2G17950 from *Arabidopsis* (AtWUS); XP007203178.1 from *Prunus persica* (PpWUS); TraesCS2A02G491900 from *Triticum aestivum* (TaWUS); KAF7004049 from *Triticum aestivum* (TaWUS); Potri.005G114700.1 from *Populus trichocarpa* (PotriWUS1). Bold red line indicates conserved domain of these proteins.

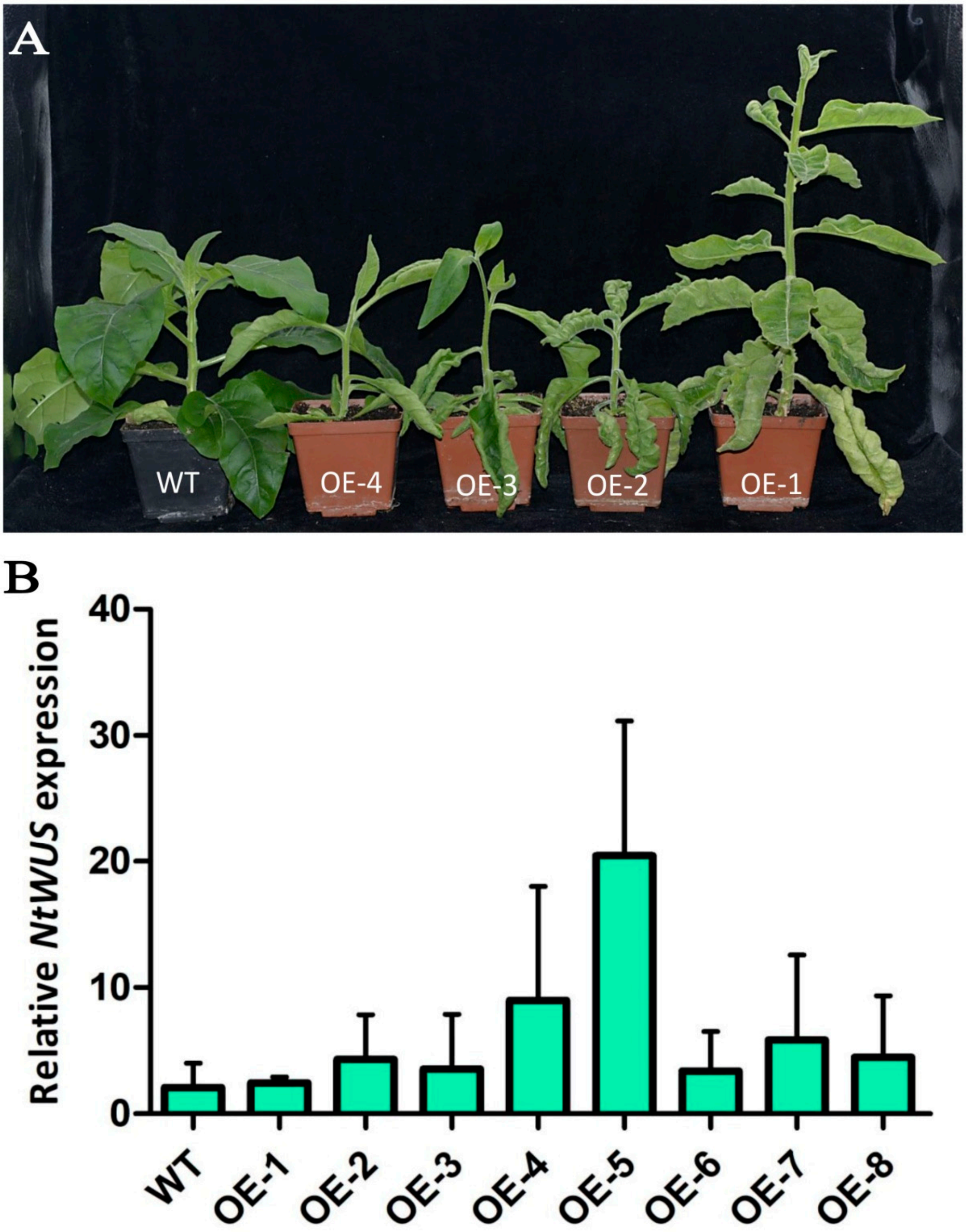

Figure S4. (A) Phenotypic analysis of some transgenic lines from *CsWUS*. (B) Relative expression of endogenous *WUS* homolog (*NtWUS*) in transgenic *CsWUS*-OE tobacco lines. *NtActin* was worked as internal control; the mean SD$\pm$ of three biological replicates is presented.

Figure S4. Tissue specific expression analysis of *CsCYCD3*. Real time PCR validation for *CsCYCD3* expression in different tissues; leaf, flower, fruit, stem, apical meristem, and root. *CsActin* was used as internal control; mean ± SD of three biological replicates is presented.

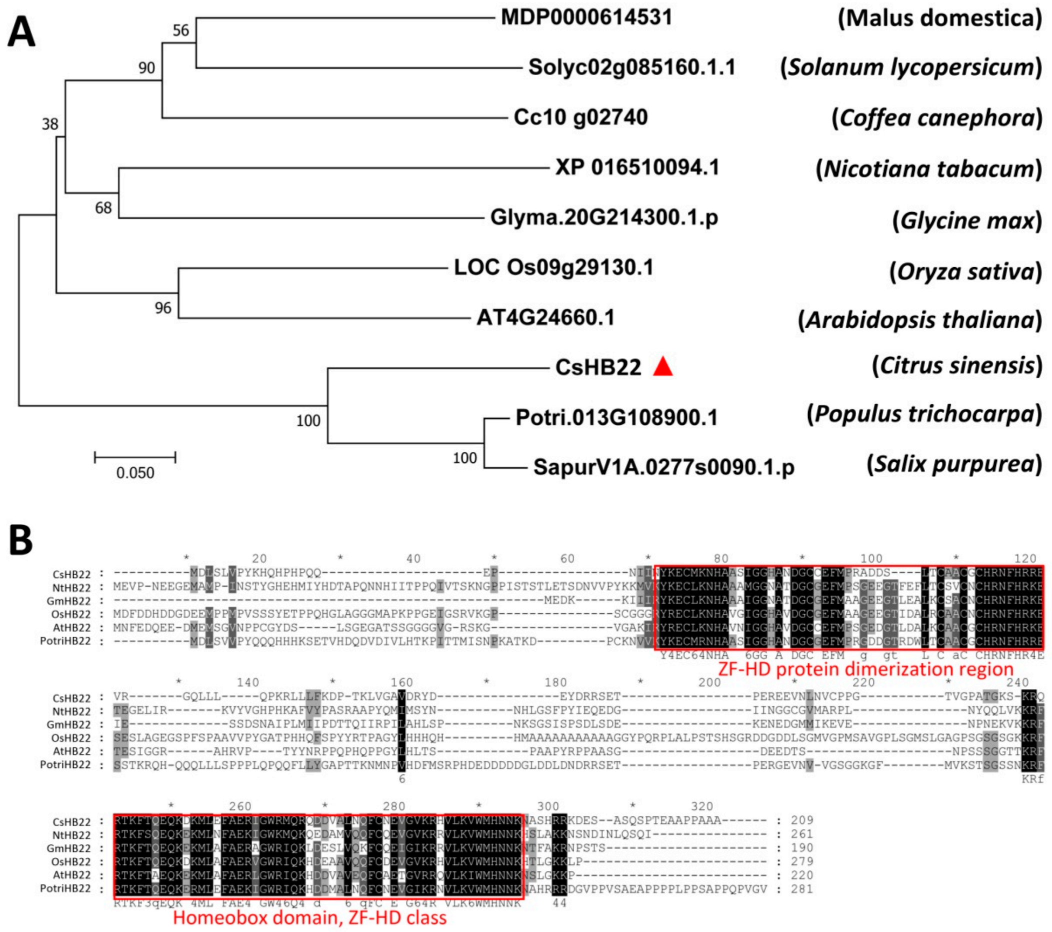

Figure S5. Phylogenetic and sequence alignment of CsHB22 with its homologs. (**A**) Phylogenetic tree of CsHB29 with its homolog proteins. (**B**) Sequence analysis of CsHB22 protein with its homolog proteins. CsHB22 from *Citrus sinensis*; LOC_Os09g29130.1 from *Oryza sativa* (LOC_Os09g2); XP_016510094.1 from *Nicotiana tabacum* (XP_0165100); Glyma.20G214300.1.p from *Glycine max* (Glyma.20G); AT4G24660.1 from *Arabidopsis* (AT4G24660); Potri.013G108900.1 from *Populus trichocarpa* (Potri.013G). Bold red line indicates conserved domain of these proteins.

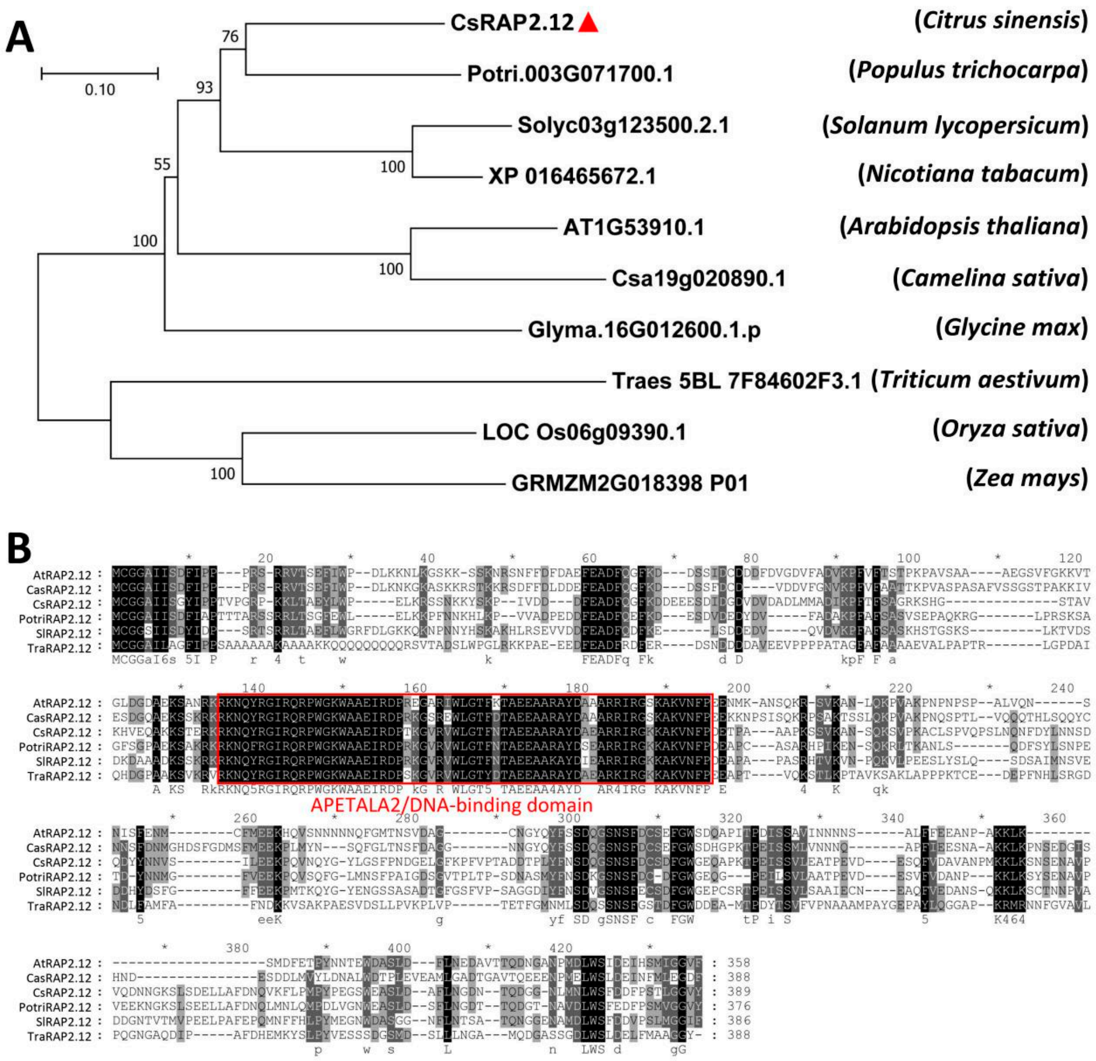

Figure S6. Phylogenetic and sequence alignment of CsRAP2-12 with its homologs. (**A**) Phylogenetic tree of CsRAP2-12 with its homolog proteins. (**B**) Sequence analysis of CsRAP2-12 protein with its homolog proteins. CsRAP2-12 from *Citrus sinensis*; Csa19g0208 from Camelina sativa (Csa19g020890.1); AT1G53910 from *Arabidopsis* (AT1G53910.1); Potri.003G from *Populus trichocarpa* (Potri.003G071700.1); Solyc03g12 from *Solanum lycopersicum* (Solyc03g123500.2.1); Traes_5BL from *Triticum aestivum* (Traes_5BL_7F84602F3.1). Bold red line indicates conserved domain of these proteins.

Supplementary Table S1. Primers used in this study.

Function	Name	Sequence 5' to 3'
Gene amplification	*CsWUS*-F	ATGGAACCTCAACAACAGCA
	CsWUS-R	TTATATAGAGCCCGAGGATC
Primer for overexpression	Pbi121-CsWUS-F	ACGGGGGACTCTAGAGGATCCATGGAACCTCAACAACAGCA
	Pbi121-CsWUS-R	CGATCGGGGAAATTCGAGCTCTTATATAGAGCCCGAGGATC
qRT-PCR	CsWUS-F	GGGGTTTCTGCTACACTTCCT
	CsWUS-R	ATGGGAAAGAGAGGGAGGGTT
	CsWOX1-F	TGACGAACACAAGACCACTA
	CsWOX1-R	CTTTCCTGTTAAATGATCATCT
	CsWOX2-F	ATAACCGCTCCTTCCTCTTC
	CsWOX2-R	ATCCTTGTCCTCCTTCTCCG
	CsWOX3-F	AGAACACCAATCTCTTGCCCTT
	CsWOX3-R	CATTTTTGCCTTCAATCTTACC
	CsWOX4-F	AACGGTTACGCCCTCTTGCT
	CsWOX4-R	GTATCCCTATCTGCTCCTGC
	CsWOX5-F	GCTGTTTATGCTATGATTTGTGAG
	CsWOX5-R	GGATTTGGAGCTGTGCAGGTGTCG
	CsWOX6-F	GCTGGCTCTTCTTCATTTTGTG
	CsWOX6-R	ATTGGTCCCCTTGGAATTTCTG
	CsWOX7-F	AGGGAGAGACAGAAGCGTCGCAAAG
	CsWOX7-R	GCATCCTCATCTTCTCTGACTCTTG
	CsWOX8-F	AAAGCAAGAGAGAGGCAAAAGC
	CsWOX8-R	GGCAGGAGGAGACTGAAAGGTG
	CsWOX9-F	TCAGCCTGCTCCTCTGTTTCC
	CsWOX9-R	ACTGGTTGTCTTGTCCATTTC
	CsWOX10-F	GGATGATGGGTTACAATGATAGT
	CsWOX10-R	AATGGTGATTTAGGCCAAAGAAG
Primer for VIGS	TRV2-CsWUS-F	GAAGGCCTCCATGGGGATCCATGGAACCTCAACAACAGCAA
	TRV2-CsWUS-R	GTCTTCGGGACATGCCCGGGGGAAGTGTAGCAGAAACCCCA
	pTRV1-F	TTACAGGTTATTTGGGCTGG
	pTRV1-R	CCGGGTTCAATTCCTTATC
	pTRV2-F	ATTCACTGGGAGATGATACGCT
	pTRV2-R	AGTCGGCCAACGCCGATCTCA
Subcellular localization	GFP-CsWUS-F	CCGTCGACCCCGGGGGTACCATGGAACCTCAACAACAGCA
	GFP-CsWUS-R	CCCTTGCTCACCATGAATTCTTATATAGAGCCCGAGGATC
Primer	ProWUS-F	ATGATGAATTGAAAAGCTTCTAGTCAAGA

for Y1H	ProWUS-R	TTAATAACCAAGAC<br>ATACAGAGCACATGCCTCGAGCTAAATA<br>AAACTAATGGGCCA
Primer for Y2H	BD-*CsWUS*-F	TCAGAGGAGGACCTGCATATGCACGCT GGACTCCCACAACT
	BD-*CsWUS*-R	CTAGTTATGCGGCCGCTGCAGCCTCT CTCGAGCTTTGTAGT
Primer for BiFC	cYFP-CsWUS-F	CCCAGGCCTACTAGTGGATCCATGGC CAAGCAACGACGG
	cYFP-CsWUS-R	ACCCTCGAGGTCGACGGATCCAAGGC CCAGCTCAGCGCA
	nYFP-CsCYCD3-F	TGGCGCGCCACTAGTGGATCCATGGC CAAGCAACGACGG
	nYFP-CsCYCD3 -R	GACAGTACTATCGATGGATCCAAGGC CCAGCTCAGCGCA
Primer For LUC	0800-proWUS1-F	AGGTCGACGGTATCGATAAGCTTCT AGTCAAGATTAATAACCAAGAC
	0800-proWUS1-R	GCCGCTCTAGAACTAGTGGATCCTCGA GACTCGAACCTTAAAA
	62sk-RAP2-12-F	GCCGCTCTAGAACTAGTGGATCCAT GTGTGGAGGTGCTATCAT
	62sk-*RAP2-12*-R	GAGGTCGACGGTATCGATAAGCTTA TACACTCCCCCTAGGGTAG
	0800-proWUS2-F	AGGTCGACGGTATCGATAAGCTTGC GCGTGCTCTACTAGTACA
	0800-proWUS2-R	GCCGCTCTAGAACTAGTGGATCCCTA AATAAAACTAATGGGCCA
	62sk-*HB29*-F	GCCGCTCTAGAACTAGTGGATCCATG GATCTCAGTTTGGTTCC
	62sk-*HB29*-R	GAGGTCGACGGTATCGATAAGCTTCG CCGCAGCCGGAGGAGCCG
Primer for motif binding	AD-*CsWUS*-F	GTACCAGATTACGCTCATATGATGGAA CCTCAACAACAGCA
	AD-*CsWUS*-R	AGCTCGAGCTCGATGGATCCTTATATA GAGCCCGAGGATC
Prime for RT-PCR	RT-*RAP2-12* F	GTGGAGGTGCTATCATTTCGGG
	RT-*RAP2-12* R	ATCGGCTTTGAGTACTTCTTGT
	RT-*HB29* F	AACCAAACATAATCAACTACAAAG
	RT-*HB29* R	GGGTCCTTGAATAGCAACAAACGC

RT-*CsCYCD3* F	GAATTGGGAGGACGAACTGA
RT-*CsCYCD3* R	ATTGGAACGGGAACTGGGAT
